# Patient Engagement With Urologists on Social Media in a Community Practice

**DOI:** 10.7759/cureus.18029

**Published:** 2021-09-16

**Authors:** Navid A Leelani, Phillip A Barnett, Stephanie Nguyen, Dustin C Hyatt

**Affiliations:** 1 Medicine, Alabama College of Osteopathic Medicine, Dothan, USA; 2 Urology, North Alabama Medical Center, Florence, USA

**Keywords:** urology, social media, community practice, kidney stones, urinary incontinence, erectile dysfunction

## Abstract

Background

Based on the Boston Area Community Health Survey, 52 million adults in the United States will have lower urinary tract symptoms, urine leakage, painful bladder syndrome, and prostatitis, which may parallel the prevalence of cardiovascular disease. In the year 2000, benign prostatic hyperplasia (BPH) accounted for 117,000 emergency department visits and 105,000 hospitalizations. These numbers underscore the burden of urological conditions and highlight the importance of patient education in preventing unnecessary hospitalizations and emergency department visits. Certain factors that may alter the progression and severity of disease include physical activity and other lifestyle changes. Based on current trends, patient education via social media may be an invaluable tool in limiting the burden on urologists and the healthcare system in the future.

Aims

This study aims to determine whether patients in a community urology practice would engage with their urologists over social media and if the likelihood to engage was associated with various demographic factors. Furthermore, the likelihood to engage actively (defined as commenting/sharing) versus passively (defined as liking a post) on two different topics within the scope of urology was determined. The two topics used were erectile dysfunction (ED)/urinary incontinence and kidney stone prevention.

Methods

Participants were recruited from a community urologic clinic in Alabama. During the month of April 2021, 293 participants completed a survey that included basic demographic questions as well as questions with a visual analog 5-point Likert scale. Responses on the Likert scale were given a value of one (very unlikely) to five (very likely), and a two-tailed Mann-Whitney U test with an α of 0.05 was used to determine significance in differences of responses. In the case of ties, the mid-rank method was used to assign ranks. For analysis of the Likert scale responses, only those respondents who had social media accounts were included.

Results

Overall, respondents were more likely to interact with a post by their urologist passively engage rather than actively. They were also less likely to passively and actively engage on a topic concerning ED/urinary incontinence versus kidney stone prevention. On the topic of kidney stone prevention, respondents were less likely to engage actively than passively. There was no difference in the likelihood of actively or passively engaging on the topic of ED/urinary incontinence. Compared to men, women were more likely to actively and passively engage on social media. On the topic of ED/urinary incontinence, women were more likely than men to actively engage; however, there was no difference in passive engagement. On the topic of kidney stone prevention, women were more likely to actively and passively engage. When looking at the likelihood of engagement based on age, there was no difference in active or passive engagement between those 55 and under or older than 55. This held true when data were stratified by topic.

Conclusion

Based on these results, the maximum impact of a social media page from a urological practice would be gained by focusing on preventative practices for less sensitive urological conditions. Furthermore, the data suggests that as the population of social media users continues to age, physicians should not expect a change in engagement patterns anytime soon.

## Introduction

As physician-patient interactions see an increased use of virtual modalities, physicians must adapt and find ways to engage with their patients and disseminate information in an effective manner. In the past decade, the use of social media has skyrocketed, with a 2016 study by Facebook showing there are 1.59 billion active Facebook users [1]. The prolific use of social media provides an avenue for physicians to engage with their patient population at large and provide a source of reliable information. Although this concept is not a novel idea, there is a limited understanding of how the current patient population in a community urology practice perceives the use of social media.

Previous studies into the use of social media in healthcare have shown some benefits. A 2020 study showed that enhanced patient education via visual aids, phone calls, mobile apps, multimedia education, and social media apps may be beneficial in the detection of colonic polyps and adenomas [2]. Furthermore, a 2014 study demonstrated the effectiveness of social networking sites such as Twitter in promoting behavioral change and its use as an educational platform for patients [3]. Based on the Boston Area Community Health Survey, 52 million adults in the United States will have lower urinary tract symptoms, urine leakage, painful bladder syndrome, and prostatitis, which may parallel the prevalence of cardiovascular disease [4]. In the year 2000, benign prostatic hyperplasia (BPH) accounted for 117,000 emergency department visits and 105,000 hospitalizations [5]. These numbers underscore the burden of urological conditions and highlight the importance of patient education in preventing unnecessary hospitalizations and emergency department visits. Certain factors that may alter the progression and severity of disease include physical activity and other lifestyle changes [6-8]. Based on current trends, patient education via social media may be an invaluable tool in limiting the burden on urologists and the healthcare system in the future and improving outcomes.

This study aims to determine whether patients in a community urology practice would engage with their urologists over social media and if the likelihood to engage was associated with various demographic factors. Furthermore, the likelihood to actively engage (defined as commenting or sharing) versus passively engage (defined as liking a post) on two different topics within the scope of urology was determined. The two topics used were erectile dysfunction (ED)/urinary incontinence and kidney stone prevention.

## Materials and methods

Participants and procedures

Participants were consecutively recruited from a urology clinic in Alabama. Only patients who were present for a clinic visit and not a procedural visit were asked to participate in the study. This did not include family members or spouses present with the patients. During the month of April 2021, 293 participants completed a survey that included basic demographic questions as well as questions with a visual analog 5-point Likert scale (see Appendix).

Data analysis

Responses on the Likert scale were given a value of one (very unlikely) to five (very likely), and a two-tailed Mann-Whitney U test with an α of 0.05 was used to determine significance in differences of responses. In the case of ties, the mid-rank method was used to assign ranks. In order for a response to be included in the data analysis, the independent and dependent variables both had to be completed on the survey. For analysis of the Likert scale responses, only those respondents who had social media accounts were included.

## Results

**Figure 1:**
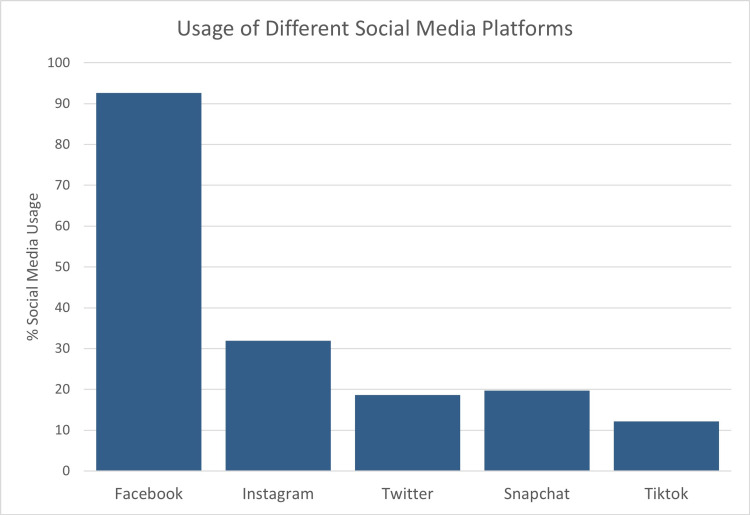
Usage of different social media platforms Social media platforms used by those respondents who had a social media account (n=188)

**Figure 2:**
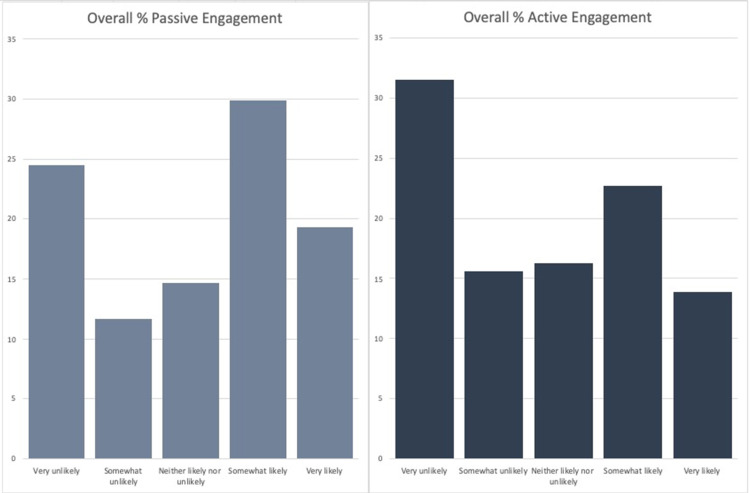
Likelihood of overall active versus passive engagement Of the respondents that had a social media account they were more likely to passively engage on any topic (n=368) versus actively engage (n=736) p-value < 0.01

**Figure 3:**
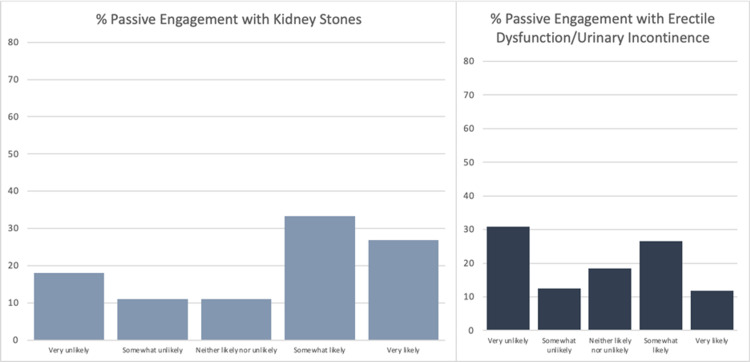
Likelihood of passive engagement for kidney stones versus erectile dysfunction/urinary incontinence Of the respondents that had a social media account they were more likely to passively engage on a topic concerning kidney stones (n=183) versus erectile dysfunction (ED)/urinary incontinence (n=185) p-value < 0.0001

**Figure 4:**
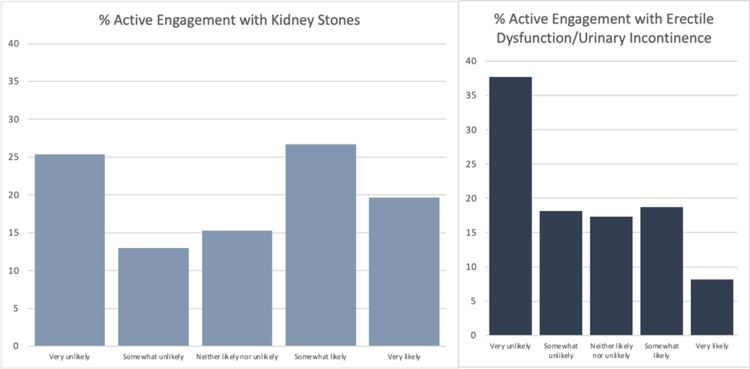
Likelihood of active engagement for kidney stones versus erectile dysfunction/urinary incontinence Of the respondents that had a social media account they were more likely to actively engage on a topic concerning kidney stones (n=367) versus erectile dysfunction (ED)/urinary incontinence (n=369) p-value < 0.0001

**Figure 5:**
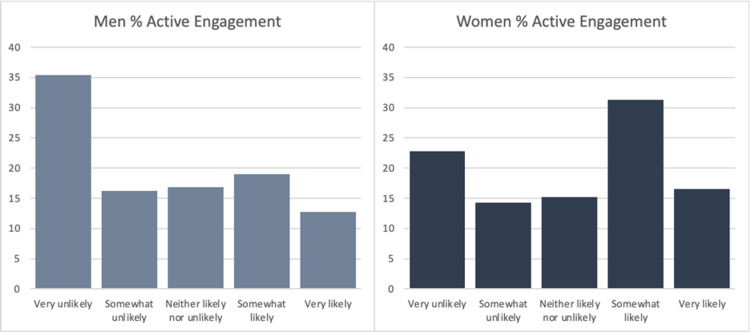
Likelihood of active engagement for men versus women Of the respondents that had a social media account, women (n=224) were more likely to actively engage on any topic in comparison to men (n=512) p-value < 0.0001

**Figure 6:**
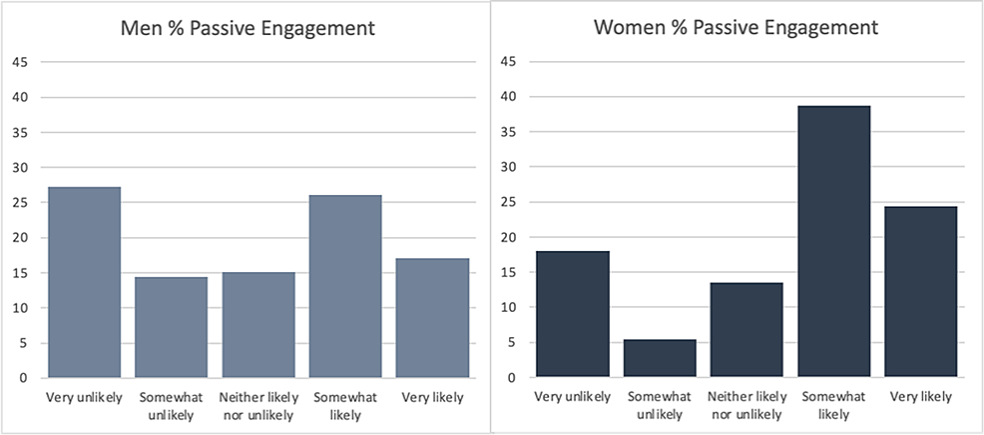
Likelihood of passive engagement for men versus women Of the respondents that had a social media account, women (n=111) were more likely to passively engage on any topic in comparison to men (n=257) p-value < 0.01

**Figure 7:**
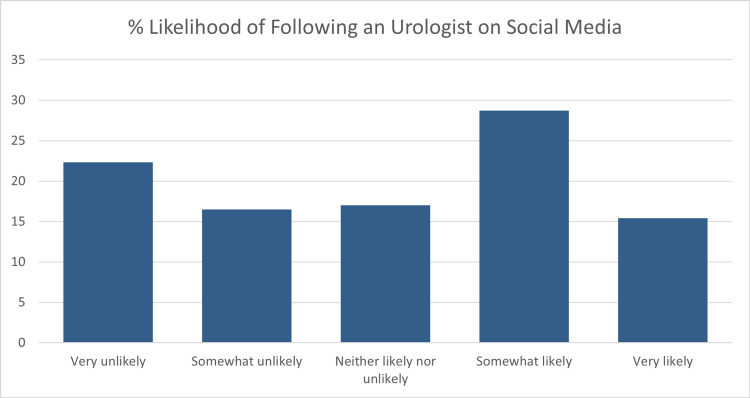
Likelihood of following a urologist on social media Percent likelihood of following a urologist on social media (n=188)

The final sample consisted of 293 responses with a response rate of 93.0%. Of the respondents, 74.1% were male, and 25.9% were female. The average age of respondents was 60.8 (n=287). 65.7% of respondents had a social media account (n=286). Figure 1 shows the breakdown of social media platforms used by those respondents who had a social media account (n=188). The following results include only those respondents who had a social media account. The average age of respondents with a social media account was 55.7. Overall, respondents were more likely to engage passively than actively with a post by their urologist (Figure 2). They were also less likely to passively and actively engage on a topic concerning ED/urinary incontinence versus kidney stone prevention (Figures 3 and 4). On the topic of kidney stone prevention, respondents were less likely to actively engage than passively engage (p-value < 0.01). There was no difference in the likelihood to actively or passively engage on the topic of ED/urinary incontinence (p-value > 0.05). Compared to men, women were more likely to actively and passively engage on social media (Figures 5 and 6). Women were more likely than men to actively engage on the topic of ED/urinary incontinence (p-value < 0.01); however, there was no difference in passive engagement (p-value > 0.05). When looking at kidney stone prevention, women were more likely to actively and passively engage (p-value < 0.01). When determining the likelihood of engagement based on age, there was no difference in active or passive engagement between those 55 and under or older than 55 (p-value > 0.05). This held true when data were stratified by topic. Respondents were also asked about their likelihood to follow their urologist on social media. Figure 7 shows the distribution of responses. Women were more likely to follow their urologist on social media than men (p-value < .05). There was no difference in likelihood to follow between those 55 and under or over 55 (p-value > 0.05). Respondents were asked about access to the internet, and 84.9% of patients reported access to the internet at home (n=292). Finally, respondents were asked about how often they would like to see their urologist post on social media, with 44.1% of respondents selecting once a month and 24.3% selecting weekly which represent the two most popular responses.

## Discussion

As the use of social media becomes more prevalent within healthcare practices, it is important to understand how patients may engage with social media posts by their physicians. Urology is unique in that the conditions addressed by urologists can be more sensitive in nature in comparison to other fields. Therefore, it is hard to gauge how patients may engage their urologists over social media since patients may have varying comfort levels when interacting with a social media post that can be viewed by others. The results of this study provide information on the potential impact that posts by urologists may have on those patients who follow them. The results show that patients were more likely to passively engage than actively engage regardless of the sensitivity of the topic, which demonstrates patient hesitancy to comment or share information on social media regarding health conditions. However, patients were more likely to actively engage in a topic concerning kidney stone prevention in comparison to something more sensitive such as ED or urinary incontinence. These results suggest having a greater proportion of posts that are less sensitive in nature can promote increased patient engagement. When looking at the predicted increase in the prevalence of urological conditions, the value of preventative healthcare cannot be understated. Therefore, the maximum impact of a social media page from a urological practice would be gained by focusing on preventative practices for less sensitive urological conditions.

It is no secret that social media use is more popular amongst younger people, and theoretically, this should correlate with increased engagement on social media. However, as the survey results show, there was no difference in the likelihood to engage over social media or likelihood to follow when stratifying by age. There are multiple factors that could be playing a role here. One factor could be the sensitive nature of topics within urology transcends age, and therefore regardless of age, patients will have similar engagement patterns. Another factor could be that younger patients who are overall healthier would be less likely to engage with healthcare-related topics on social media. Regardless, this shows that as the population of social media users continues to age, physicians should not expect a change in engagement patterns.

Another barrier to social media engagement is a lack of internet access, particularly in community practices with no large medical centers in the area. A 2018 census study found that 85.2% of urban households in the southern United States have internet [9]. This stood true based on the survey results, which showed that 84.9% percent of respondents had access to the internet at home. As the population continues to age, this number should continue to rise as the same census showed that younger populations have more access to the internet. This increased internet access in the future urological patient population may drive increased engagement over social media.

As the increased burden of patients with urological issues looms closer, it is vital that community urologists educate their patient population on preventative measures. Increasingly, urologists are using social media to interact with other urologists in an academic and collegial manner [10]. It would not take much more effort to engage their patients, and based on the survey results; this could be as simple as a single post on social media a month to keep patients engaged. Furthermore, the use of social media to engage with patients may provide an avenue to reach a broader audience and potentially create a “virtual relationship” with the patient that may develop into an office visit that otherwise would have been avoided, potentially leading to improved patient outcomes and compliance. As the patient-physician relationship continues to evolve due to technological advances, so should the way physicians interact with their patients.

Limitations

Limitations of this study include response bias due to respondents avoiding the extreme options or answering questions in a particular way to be viewed more favorably. Although, every respondent was told that this study was anonymous. Another limitation is the fatigue bias commonly seen on Likert scale surveys in which respondents select choices just to complete the survey, and the responses do not reflect their true feelings. Finally, another potential limitation is selection bias due to the substantial difference between the number of male and female participants. 

## Conclusions

Overall, respondents were more likely to engage passively than actively with a post by their urologist. They were also less likely to passively and actively engage on a topic concerning ED/urinary incontinence versus kidney stone prevention. On the topic of kidney stone prevention, respondents were less likely to actively engage than passively engage. There was no difference in the likelihood to actively or passively engage on the topic of ED/urinary incontinence. Compared to men, women were more likely to actively and passively engage on social media. When looking at the likelihood of engagement based on age, there was no difference in active or passive engagement between those 55 and under or older than 55. This held true when data were stratified by topic. Facebook is currently the most widely used social media platform.

Algorithms developed by the different social media platforms are used to determine what social media posts are seen by individual users. Although the algorithms are not fully understood, what is known is that if an individual engages with a post, they are more likely to see posts in the future from that same page as well as similar posts. Therefore, in order to increase the odds of patients seeing future posts on social media by their urologist, they would need to engage with an initial post. Based on these results, the maximum impact of a social media page from a urological practice would be gained by focusing on preventative practices for less sensitive urological conditions. Furthermore, the data suggests that as the population of social media users continues to age, physicians should not expect a change in engagement patterns anytime soon.

## Appendices

1.     Age____________________

2.     Gender

a.      Male

b.     Female

c.      Other (Please specify)___________________

3.     Annual Income

a.      0-$50,000

b.     $51,000-$100,000

c.      $101,000-$150,000

d.     $151,000-$200,000

e.     $200,000+

4.     Access to internet at home?

a.      Yes

b.     No

5.     Do you have a social media account if so circle all that apply

a.      Do not have a social media account

b.     Facebook

c.      Instagram

d.     Twitter

e.     Snapchat

f.       Tik-Tok

g.      Other (Please specify)______________________

6.     How likely would you be to follow your Urologist on social media

     O----------------------O----------------------O----------------------O----------------------O

Very                        Somewhat           Neither likely            Somewhat               Very

unlikely                  unlikely                 nor unlikely               likely                         likely  

7.     How likely would you be to “Like” a post by your Urologist on social media on a topic such as Erectile Dysfunction or Urinary Incontinence

     O----------------------O----------------------O----------------------O----------------------O

Very                        Somewhat           Neither likely            Somewhat               Very

unlikely                  unlikely                 nor unlikely               likely                         likely  

8.     How likely would you be to “Comment” on a post by your Urologist on social media on a topic such as Erectile Dysfunction or Urinary Incontinence

     O----------------------O----------------------O----------------------O----------------------O

Very                        Somewhat           Neither likely            Somewhat               Very

unlikely                  unlikely                 nor unlikely               likely                         likely  

9.     How likely would you be to “Share/Retweet” a post by your Urologist on social media on a topic such as Erectile Dysfunction or Urinary Incontinence

     O----------------------O----------------------O----------------------O----------------------O

Very                        Somewhat           Neither likely            Somewhat               Very

unlikely                  unlikely                 nor unlikely               likely                         likely  

10. How likely would you be to “Like” a post by your Urologist on social media on a topic such as Kidney Stone Prevention

     O----------------------O----------------------O----------------------O----------------------O

Very                        Somewhat           Neither likely            Somewhat               Very

unlikely                  unlikely                 nor unlikely               likely                         likely  

11. How likely would you be to “Comment” on a post by your Urologist on social media on a topic such as Kidney Stone Prevention

     O----------------------O----------------------O----------------------O----------------------O

Very                        Somewhat           Neither likely            Somewhat               Very

unlikely                  unlikely                 nor unlikely               likely                         likely  

12. How likely would you be to “Share/Retweet” a post by your Urologist on social media on a topic such as Kidney Stone Prevention

     O----------------------O----------------------O----------------------O----------------------O

Very                        Somewhat           Neither likely            Somewhat               Very

unlikely                  unlikely                 nor unlikely               likely                         likely  

13.  How often would you like to see your Urologist post on social media

a.      Never

b.     Once a year

c.      Once a month

d.     Once a week

e.     Daily
